# Safety Aspect of* Enterococcus faecium* FL31 Strain and Antibacterial Mechanism of Its Hydroxylated Bacteriocin BacFL31 against* Listeria monocytogenes*

**DOI:** 10.1155/2018/5308464

**Published:** 2018-11-01

**Authors:** Ahlem Chakchouk-Mtibaa, Imen Sellem, Yosra Kamoun, Slim Smaoui, Ines Karray-Rebai, Lotfi Mellouli

**Affiliations:** ^1^Laboratory of Microorganisms and Biomolecules, Center of Biotechnology of Sfax, University of Sfax, Road of Sidi Mansour Km 6, P.O. Box 1177, 3018, Tunisia; ^2^Laboratory of Molecular Biotechnology of Eukaryotes, Center of Biotechnology of Sfax, University of Sfax, Road of Sidi Mansour Km 6, P.O. Box 1177, 3018, Tunisia

## Abstract

In previous work we have isolated and identified a new strain called* Enterococcus faecium* FL31. The active compound secreted by this strain, “BacFL31”, has been purified and characterized. In the present study, safety aspect, assessed by microbiological and molecular tests, demonstrated that* Enterococcus faecium* FL31 was susceptible to relevant antibiotics, free of hemolytic, gelatinase, DNase, and lipase activities. In addition, it did not harbor virulence and antibiotic resistance genes. Combined SYTOX Green dye and UV-absorbing experiments, along with released extracellular potassium and transmembrane electrical potential measurements, showed that pure BacFL31 at a concentration of 1×MIC (50 *μ*g/mL) could damage cytoplasmic membrane of the pathogen* Listeria monocytogenes* ATCC19117. The same concentration causes the leakage of its intracellular constituents and leads to the destruction of this pathogenic microorganism. In summary, this work reflected characteristics of* Enterococcus faecium* FL31 strain and its bacteriocin in terms of functional and safety perspectives.

## 1. Introduction

*Enterococcus* is a large genus of lactic acid bacteria (LAB) and several species from this genus have been used as probiotic for humans or animals [1]. In addition, some* Enterococcus faecium* spp. act as protective agents against food-spoilage and pathogenic bacteria, such as* Listeria monocytogenes*,* Salmonella typhimurium*,* Staphylococcus aureus,* and* Clostridium perfringens* spores due to their ability to produce antimicrobial peptides called bacteriocins (enterocins) [2–5].

However, certain species of* Enterococcus faecium* can have relatively low virulence and cause nosocomial infections especially endocarditis, septicemia, urinary tract infections, meningitis, and others human infections [6]. These pathogenic strains can also carry multiple antibiotic resistances and several virulence factors like haemolysin, gelatinase, invasins, adhesins, cytolysin, and enterococcal surface proteins [7]. It should be noted that several studies have shown that enterococci possessing virulence genes are only isolated from infected patients and clinical samples, whereas the majority of* Enterococcus* strains isolated from foodstuffs have probiotic effects and health benefits [2]. In food storage, the application of bacteriocins of LAB as natural preservatives to control the growth of spoilage and pathogenic bacteria in food requires the safety confirmation of the producing strain and the understanding of its bacteriocin action mechanism against food-spoilage and pathogenic bacteria [8].

In previous works, a strain called FL31, isolated from fermented olives, was selected for its antimicrobial activity and identified a new lactic acid bacteria designated* Enterococcus faecium* FL31. The active compound of the strain FL31 was identified as a proteinaceous substance and named BacFL31. The N-terminal amino acid sequence of the purified BacFL31 showed the presence of hydroxyproline residues. BacFL31 exhibits a bactericidal mode of action against* Listeria monocytogenes* ATCC19117 and was proved to be useful for the inhibition of the growth of this pathogen during storage at 4°C of minced beef meat [9].

Taking into account the attractive characteristic of the* Enterococcus faecium* FL31 strain and its original bacteriocin BacFL31, we propose in the present paper, to define the probiotic properties and the safety of this strain as well as the elucidation of the bactericidal mechanism of BacFL31 against* L. monocytogenes *ATCC19117.

## 2. Materials and Methods

### 2.1. Bacterial Strains, Media, and Growth Conditions

*E. faecium* FL31, BacFL31 producer strain [9], was grown in a De Man, Rogosa, and Sharpe (MRS) broth medium at 37°C for 18 h [10].* Enterococcus faecalis* ATCC 29212 was grown overnight at 37°C in Brain Heart Infusion (BHI). The genomic DNA of this strain was extracted using molecular biology kit (Bio Basic Canada Inc.) and used as a positive control for the evaluation of the pathogenicity of* E. faecium* FL31.* Staphylococcus aureus* ATCC 6538 was cultured in LB medium overnight at 37°C and used as a positive control to study DNase and lipase activities. To measure the BacFL31 activity and to study its mechanism of action, we used the food-borne pathogen* L. monocytogenes* ATCC 19117 as target strain. This microorganism was cultured in Luria-Bertani (LB) medium overnight at 30°C.

### 2.2. Safety Evaluation of Bacteriocinogenic* E. faecium* FL31

#### 2.2.1. Antibiotic Resistance

The susceptibility of the strain* E. faecium* FL31 to a range of relevant clinically most used antibiotics (*μ*g/disc): ampicillin (30), streptomycin (10), kanamycin (30), chloramphenicol (30), tetracycline (30), spectinomycin (100), penicillin G (30), vancomycin (30), oxacillin (5), and amoxicillin (25) were tested by disk diffusion method on MRS solid media. Antibiotics discs were applied on MRS plates containing 10^7^ CFU of* E. faecium* FL31 spread uniformly across the surface and then plates were incubated at 37°C for 24 h. Inhibition zones around the discs were measured in mm and the results were interpreted following the criteria of the Antibiogram Committee of the French Microbiology Society CA-SFM [11].

#### 2.2.2. Hemolytic Activity, Gelatinase, DNase, and Lipase Tests

For hemolytic activity, fresh culture of* E. faecium* FL31 was streaked on Columbia agar plates containing 5% (w/v) sheep blood and incubated for 48 h at 37°C. Blood agar plates were examined for signs of *β*-hemolysis (clear zones around colonies).* E. faecalis* ATCC 29212 were used as a positive control for *β*–hemolysis assay.

Gelatinase activity of the strain* E. faecium* FL31 was assessed according to Su et al., 1991 [12]. GelE-positive colonies on gelatine medium were surrounded by a turbid halo after 2 days of incubation at 37°C. To measure the hydrolyzed gelatine in the agar plates, 0.5-1.0 mL of Frazier solution (mercuric chloride, 15.0 g; hydrochloric acid (37%), 20 mL; distilled water, 100 mL) was poured on the surface of the medium to precipitate the unhydrolyzed gelatine.* E. faecalis* ATCC 29212 was used as a positive control.

DNase activity was tested using DNase agar medium [13]. The plate was inoculated with the appropriate strain by streaking a thick line of inoculum across the plate. After incubation at 37°C for 24-48 hours, the surface of the DNase test agar plate was flooded with Toluidine Blue solution. DNase activity is indicated by a pink zone surrounding growth. The color of the medium remains unchanged if the test is negative.* S. aureus* ATCC 6538 was used as a positive control.

Lipase activity was performed according to Tiago et al., 2004 [14]. The appropriate strain was inoculated in MLB (tryptone 1%; 0.5% yeast extract; 0.5% NaCl) agar supplemented with 2.0 g/L of CaCl2 and 10 g/L of Tween-80. Plate was incubated at 37°C for 24-48 hours. A positive reaction was indicated by a clear halo around the colonies.* S. aureus* ATCC 6538 was used as a positive control.

#### 2.2.3. Detection of Virulence Genes in* E. faecium* FL31 Strain

The absence or the presence of different virulence genes in* E. faecium* FL31 was evaluated by PCR using specific primers (Table 1). The tested genes were* asa1* (aggregation substance),* ace* (adhesin of collagen protein),* esp* (enterococcal surface protein),* efaAfm* (cell wall adhesin), and* cylB* (activation and expression of cytolysin). The oligonucleotide primers were purchased from Bio Basic Canada Inc.

PCR amplifications were performed on MultiGene™ OptiMax Thermal Cycler with a final volume of 25 *μ*L reaction mixtures containing 5x Dream-Taq reaction buffer, 50 ng of bacterial DNA (2*μ*L of the stock), 100 *μ*M DNTP, 25 pM of each primer, and 1U of Taq DNA polymerase (Dream-Taq). PCR amplification of target genes was carried out using a program consisting of the initial denaturation at 95°C for 5 min, followed by 30 cycles of denaturation at 94°C for 1 min, annealing at an appropriate temperature for 1 min, elongation at 72°C for 1 min, and a final extension step of 7 min at 72°C. The DNA from strain* E. faecalis* ATCC 29212 (clinical pathogen) was used as positive control. PCR products were resolved by electrophoresis in 1% agarose (Mupid EXU Japan) gels and digitized by the UVP VisiDoc-It™ Imaging System, Upland, CA, USA.

### 2.3. Targeting Bacteriocin Genes

Total genomic DNA of* E. faecium* FL31 was used as template for the detection of some enterocins genes (*Ent B*,* Ent A*,* Ent P*,* Ent* L50*A,* and* Ent* L50*B*) encoding known* Enterococcus *bacteriocins. The latter's specific primers and PCR conditions were described in Table 2.

PCR amplifications were performed in a final volume of 50 *μ*L containing 50 ng of bacterial DNA, 5x Dream-Taq reaction buffer, 200 *μ*M DNTP, 50 pM of each primer, and 1U of Taq DNA polymerase (Dream-Taq).

The following PCR conditions were used: denaturation at 94°C for 4 min, followed by 35 cycles of denaturation at 94°C for 1 min; annealing at an appropriate temperature depending on Tm of each primer pair for 1 min; elongation at 72°C for 1 min and a final extension step of 7 min at 72°C. The PCR generated fragments were analyzed by electrophoresis in 1. 2% agarose gels.

### 2.4. BacFL31 Purification and Determination of Minimal Inhibitory Concentration (MIC)

BacFL31 was purified as described by Chakchouk-Mtibaa et al., 2014 [9]. Strain FL31 was inoculated in 1% v/v into 900 ml of MRS broth and incubated without agitation at 37°C until early stationary phase (18 h) corresponding to a maximum of bacteriocin production level. Four steps were used to purify BacFL31 from the obtained active supernatant. Briefly, the first step involved the heat treatment of the supernatant for 15 min at 90°C and then cooling at room temperature before pelleting the denatured proteins by centrifugation at 4500 g for 30 min. The active supernatant was applied (second step) to ammonium sulphate precipitation at 60%. After refrigerated centrifugation at 9000* g* for 30 min, the precipitate was dissolved in 10 mL of 20 mmol/L sodium phosphate buffer (pH 7.0) and loaded on a column (128 X 2 cm) of gel filtration Sephadex G-25 (the third step) equilibrated with 20 mmol/L sodium phosphate buffer (pH 7). Ninety fractions (F1-F90) with 5 mL each were eluted from the Sephadex G-25 column. These fractions, detected at 280 nm, were fractioned into seven samples (S1-S7) and only the sample S2 (F38-F42) was noted to exhibit antibacterial activity against* L. monocytogenes* ATCC19117. S2 was submitted to HPLC purification (fourth step) and BacFL31 was eluted from the column with two mobile phases: A (99.9% water, 0.1% trifluoroacetic acid “TFA”) and B (99.9% acetonitrile, 0.1% TFA). The pooled biological active fraction was concentrated and stored at −20°C. BacFL31 concentration was measured using bovine serum albumin (BSA) as reference as described by Bradford in 1976 [24].

Minimal inhibitory concentration (MIC) of the pure BacFL31 against* L. monocytogenes* ATCC 19117 was determined in Mueller–Hinton broth. The test was performed in sterile 96-well microplates with a final volume in each microplate well of 100 *μ*L. A stock solution of 1.2 mg/mL of BacFL31 was twofold serially diluted in LB medium. To each test well, 10 *μ*L of* L. monocytogenes* ATCC 19117 cell suspension at 10^6^ CFU/mL was seeded. The plates were then incubated overnight at 37°C. The MIC was defined as the lowest pure BacFL31 concentration at which the microorganism does not demonstrate visible growth after incubation. Twenty-five *μ*l of Thiazolyl Blue Tetrazolium Bromide (MTT) at 0.5 mg/mL was added to the wells and incubated at room temperature for 30 min. The colorless tetrazolium salt acts as an electron acceptor and was reduced to a red-colored formazan product by the indicator microorganism. When microbial growth was inhibited, the solution in the well remained clear after incubation with MTT. Ampicillin was used as standard and experiments were performed in triplicate.

### 2.5. Membrane Permeabilization Assay

The membrane permeabilization assay of BacFL31 towards the cytoplasmic membrane of the targeted bacterial cells was carried out using SYTOX Green fluorescent dye (Thermo Fisher scientific). This molecular probe with a high affinity to DNA is unable to enter into an intact cell unless the membrane integrity is compromised by the addition of membrane-disrupting compounds.

The indicator strain* L. monocytogenes* ATCC 19117 was grown in LB broth overnight at 37°C. After centrifugation at 7000* g* for 10 min, the bacterial pellets were washed 3 times with 10 mM sodium phosphate buffer (pH 7.2) before resuspending in the same buffer to reach an optical density of 0.5 at 600 nm. Five *μ*M of SYTOX Green was added to the cells suspension. Then, 100 *μ*L of this mixture was transferred to a 96 well PCR plate. The purified bacteriocin BacFL31 (1 X MIC) was added to the mixture (bacterial cells + SYTOX® Green). The positive control contains 70% of ethanol solution instead of BacFL31. Two negative controls were used containing bacterial cells and SYTOX Green without BacFL31 or added by a solution of tetracycline at a concentration of 1.5*μ*g/mL. Tetracycline is an antibiotic that does not act on the cytoplasmic membrane. Then the 96 well PCR plate was immediately placed into a Varioskan Flash Spectral Scanning Multimode Reader (Thermo Fisher Scientific). The fluorescence signal produced by binding of SYTOX Green dye with the nucleic acid of the dead bacterial cells was detected at 520 nm for a period of 60 min. The experiment was performed in triplicate and raw data was analyzed using Microsoft Excel software.

### 2.6. Measurement of UV-Absorbing Materials

Release of UV-absorbing materials is an index of cell lysis and nonselective pore formation [25]. Absorbances were measured by UV spectrophotometer (UV-1600 PC spectrophotometer VWR) as follows:* L*.* monocytogenes* ATCC 19117 cells were resuspended in phosphate buffer 10 mM (pH 7.0) to reach a cell concentration of 10^7^ CFU/mL. After that, BacFL31 was added to the cell suspensions (10 mL) to obtain final concentrations of 1 X MIC. A control sample (10 mL of suspensions cells without bacteriocin) was used for comparison. Aliquots of 1 mL from control and BacFL31-treated cells suspensions were taken every 30 min. Cells were harvested by centrifugation (8000* g*, 10 min) and the supernatants were filtered by sterile nitrate cellulose membrane (0.22 *μ*M). The UV-absorbing material was measured at 260 and 280 nm.

### 2.7. Scanning Fluorescent Microscopy Analysis

In order to determine membrane damage of* L. monocytogenes* ATCC 19117 cells caused by BacFL31 treatment, we carried out fluorescence studies using the SYTOX Green probe (ThermoFisher scientific). The SYTOX Green was unable to enter into cell unless if the membrane integrity was compromised [26]. Two hundred *μ*L of cells suspension (10^7^ CFU/ml of* L. monocytogenes* ATCC 19117) was treated with BacFL31 at a final concentration of 1 X MIC. As control, 200 *μ*L of cells suspension without BacFL31 was used. The two mixtures, control and treated sample, were incubated with 2 *μ*L SYTOX Green (10 *μ*M final concentration) for 15 min, 30 min, and 1 h at room temperature in the dark. For each incubation time, an aliquot of cells mixture was washed with 1 mL sterile phosphate buffer (pH 7.0) and centrifuged twice at 8000* g* for 10 min. The cells fixed between a slide and quartz cover-slip were examined under a fluorescent microscope OLYMPUS DP70 digital camera connected to a TV adapter.

### 2.8. Measurement of the Released Potassium Ions

To determine the BacFL31 impact on cells membrane permeability, the released extracellular ions K+ were measured.* L*.* monocytogenes *ATCC 19117 cells were centrifuged and resuspended in sterile water to reach 10^7^ CFU/mL of cell concentration. BacFL31 was added at a final concentration of 1 X MIC to the cells suspensions. After various time intervals (15, 30, 45, 60, and 75 min), the control (without BacFL31 addition) and the treated samples (2 mL for each essay) were centrifuged at 7000* g* for 10 min. The supernatants were subjected to measurement of the released potassium ions by an Analyst 200 atomic absorption spectrometer (Perkin Elmer).

### 2.9. Determination of the Transmembrane Electrical Potential (Δ*ψ*)

The effect of BacFL31 on the proton motive force (PMF) of cytoplasmic membrane was studied by ΔΨ measurement. ΔΨ was monitored with the fluorescent probe 3,3-dipropylthia-dicarbocyanine iodide “DISC3(5) Sigma–Aldrich” [27]. This probe measures the electrical potential gradient disruption across the cytoplasmic membrane cells. The* L. monocytogenes* ATCC 19117 cells suspension (10^7^ CFU/mL) was prepared and mixed with 5*μ*M DISC3(5) and then supplemented with nigericin (Sigma–Aldrich) at 1*μ*M (negative control) or with valinomycin (Sigma–Aldrich) at 1*μ*M (positive control) or with BacFL31 at 1 X MIC. A* L. monocytogenes* ATCC 19117 cells suspension (10^7^ CFU/mL) without any addition was used as control. Fluorescence value measurements were determined with a Varioskan Flash Spectral Scanning Multimode Reader (Thermo Fisher Scientific) and the excitation and emission wavelengths were set at 622 and 670 nm, respectively.

## 3. Results and Discussions

### 3.1. Safety Evaluation of the Strain* E. faecium* FL31

#### 3.1.1. Antibiotic Susceptibility Testing

*E. faecium *FL31 was susceptible to ampicillin, streptomycin, kanamycin, chloramphenicol, tetracycline, spectinomycin, penicillin G, vancomycin, oxacillin, and amoxicillin (Table 3). The strain* E*.* faecium* MMZ01 isolated from artisanal Tunisian fermented meat [28] has a similar antibiotic susceptibility profile to* E. faecium* FL31. In the last decade, vancomycin-resistant Enterococci (VRE) became a major hospital acquired pathogen which has emerged as a frequent cause of nosocomial infections. The studied strain* E. faecium* FL31, isolated from food, was vancomycin-sensitive bacteria. This result is in agreement with many studies demonstrating that the majority of enterococci isolated from food were sensitive to this antibiotic [4].

#### 3.1.2. Physiological Test

*(1) Hemolytic Activity*. The hemolytic activity is associated with haemolysin production. It is an extracellular cytotoxic protein implicated in enterococcal virulence. The production of this protein can increase the severity of the infection and the frequent virulence factor [29]. In our case, no hemolytic activity was observed for the strain* E. faecium* FL31. This finding is in accordance with previous studies indicating that* Enterococcus *strains isolated from fermented food products exhibited no hemolytic activity [30].

*(2) Gelatinase Activity*. Gelatinase activity was not detected in* E. faecium* FL31. The same result was reported for* E. faecium *Y31 [31]. Gelatinase is a zinc metalloprotease, encoded by* gelE* that is capable of hydrolyzing gelatine, collagen, casein, haemoglobin, and other peptides [32]. As encoded by a plasmid gene, gelatinase could mediate binding to the host epithelium and it appears that it plays also an important role in promoting bacterial aggregation during conjugation, facilitating plasmid exchange [33].

*(3) DNase Activity*. Deoxyribonucleases, enzymes that hydrolyze nucleic acids to yield oligonucleotides, are involved in bacterial virulence. The strain* E. faecium* FL31 is devoid of DNase activity. Similarly, it has been reported that* E. faecalis* and* E. faecium *isolates do not produce DNase activity [34].

*(4) Lipase Activity*. The biological role of lipases might be considered the most important step in many bacterial infections [35]. The production of this enzyme enhances adhesion by degrading surface molecules of the host. According to our study, the strain* E. faecium* FL31 did not exhibit lipolytic activity.

#### 3.1.3. Screening for the Presence of Virulence Genes

The safety evaluation of* E. faecium* FL31 was investigated by the screening of genes encoding different virulent factors cited in Table 1.

**Figure 1:**
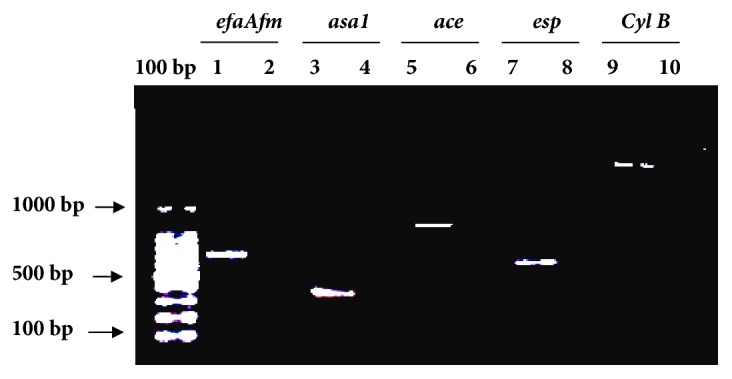
PCR screening for the presence of virulence genes in* E. faecium* FL31. Lanes 1, 3, 5, 7, and 9: amplification products of corresponding genes obtained from genomic DNA of the positive control* E. faecalis* ATCC 29212; lanes 2, 4, 6, 8, and 10: amplification products of corresponding genes obtained from genomic DNA of* E. faecium* FL31.

As shown in Figure 1,* E. faecium* FL31 strain did not harbor the five tested virulence genes* efaAfm* (705 bp),* asa1* (375 bp),* esp* (510 bp),* ace* (1008 bp), and* cylB* (2020 bp). Amplification products of these virulent genes were observed only for the positive control strain* E. faecalis* ATCC 29212 (Figure 1). In* E. faecium* FL31, the absence of genes encoding endocarditis antigen (*efaAfm*), aggregation substance (*asa1*), adhesion of collagen (*ace*), enterococcal surface protein (*esp*), and cytolysin toxic protein (*cylB*) is in agreement with the results of Ahmadova et al. [4] and Liu et al. [31], who report the absence of these virulence determinants in* E. faecium* AQ71 and* E. faecium* Y31, respectively. Eaton and Gasson (2001) reported the presence of these virulent factors only in clinical* E. faecium* strains [17].

### 3.2. PCR Amplification of Bacteriocin Genes

PCR amplification of five well-known structural enterocin (A, B, P, Q and L050) genes was used for the screening of bacteriocin genes in chromosomal and plasmidic DNA of* E. faecium* FL31 strain (Table 2). Obtained PCR products showed amplification for only enterocin B from chromosomal DNA.

Gene cluster required for enterocin production may be either associated with a plasmid or located in chromosomal DNA [20]. In many cases, enterocin production is a plasmid-encoded trait such as enterocin L50A and enterocin L50B which are located in the 50 kb plasmid pCIZ1 and enterocin Q located in the 7.4 kb plasmid pCIZ2 [36]. However enterocin genes have been also found on the chromosome such as enterocin A, enterocin B, and enterocin P [37].

Enterocin produced by enterococci, including* E. faecium* and* E. faecalis,* generally belongs to Class II bacteriocins [38]. Enterocin that comes under Class IIa contains a pediocin-like structure with a YGNGVXC amino acid motif at their N-terminus and shows strong antilisterial activity. They include enterocin A and enterocin P [20]. Enterocin B is not pediocin-like but is similar to Class IIa bacteriocins with respect to its chemical characteristics, heat stability, and anti-*Listeria* activity [39]. Several studies showed that the enterococcal strains possess one or more enterocin structural gene(s). In the study of Gutiérrez et al.,* E. faecium* P13 possess genes coding enterocins P, enterocin A, and L50A/B [21].

### 3.3. Membrane Permeabilization Test

We reported previously that BacFL31 is the first bacteriocin described as encompassing hydroxyproline residues [9]. This particular and original characteristic prompted us to study the mode of action of BacFL31 against the pathogenic* L*.* monocytogenes* ATCC19117. BacFL31 was purified to homogeneity from a cell-free culture supernatant of* E. faecium* FL31 strain as described by Chakchouk-Mtibaa et al. [9], and minimal inhibitory concentration (MIC) of the pure BacFL31 against* L. monocytogenes *ATCC19117 has been determined and is equal to 50 *μ*g/mL.

**Figure 2:**
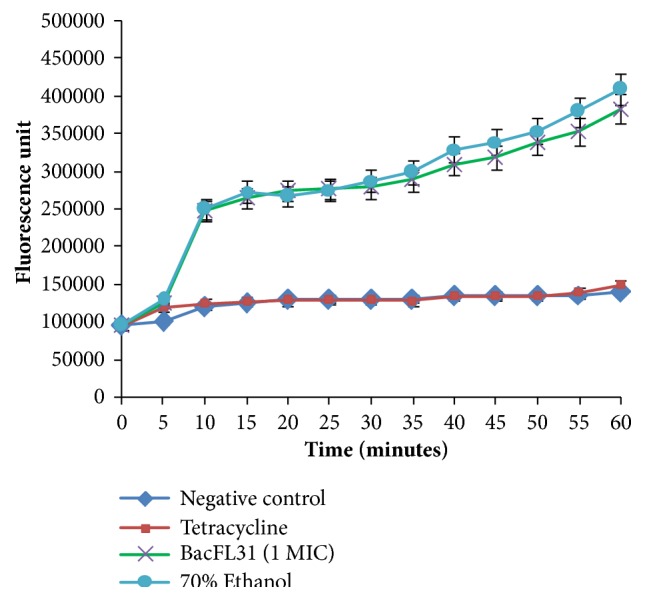
Membrane permeabilization assay of Bacteriocin Bac FL31 towards cytoplasmic membrane of* L. monocytogenes* ATCC 19117. Negative control without BacFL31 addition; positive control ethanol at 70%. Tetracycline did not show any permeability activity in this test.

The ability of the pure peptide BacFL31 to penetrate the cytoplasmic membrane of the pathogen* L. monocytogenes *ATCC 19117 was tested with SYTOX Green dye. This molecular probe is a high affinity nucleic acid stain that easily penetrates cells with compromised plasma membranes and will not cross the membranes of intact cells [26]. As shown in Figure 2, the fluorescence intensity evolution was very similar for sample treated with BacFL31 at 50 *μ*g/mL and sample treatment with a solution of ethanol at 70% (ethanol is known to destroy cell walls membranes). This fluorescence intensity increased rapidly for the first ten minutes from 100.000 to 250.000 units. Then, the fluorescence continues to increase moderately to reach approximately 420.000 units after 60 min. Concerning the two negative controls (bacterial cells and SYTOX Green without BacFL31or added by a solution of tetracycline at 1.5*μ*g/mL) no evolution of fluorescence was observed from the beginning to the end of the experiment. It should be noted that tetracycline is an antibiotic acting on protein synthesis by inhibiting translation. It binds to the 16S part of the 30S ribosomal subunit and prevents the amino-acyl tRNA from binding to the A site of the ribosome.

### 3.4. Measurement of UV-Absorbing Materials

**Figure 3:**
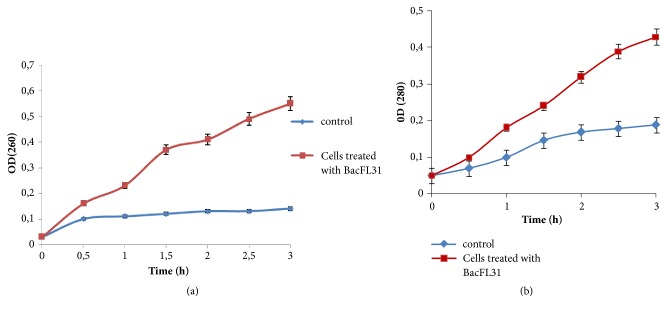
Extracellular UV-absorbing materials from* L. monocytogenes* ATCC 19117 cells detected at 260 nm (a) and 280 nm (b).

The UV-absorbing materials were measured as an index of cell lysis and nonselective pore formation. As shown in Figure 3, the absorbance value of extracellular nucleic acids (OD_260_ nm) of* L. monocytogenes* ATCC 19117 cells treated with BacFL31 increased significantly (*p<*0.05) from 0.03 to 0.55 whereas, for the negative control sample, the absorbance remains constant until the end of the experience. Absorbance value of proteins (OD_280_ nm) of BacFL31 treated cells reaches 0.42 after 3 h of incubation. A slight increase of this absorbance was noted for the negative control to reach a value of 0.15 at the end of the experiment (Figure 3). These results supported the idea that BacFL31 damages cytoplasmic membrane and causes subsequent leakage of intracellular constituents. Thus, the crucial effect of BacFL31 on* L. monocytogenes* ATCC19117 cells could be the formation of nonselective pores in the plasma membrane. Similar results have been observed with other bacteriocins such as Bifidocin A against* E. coli *1.90 [25], Bac C1 against* B. cereus* [40], and sakacin C2 against* E*.* coli* [8].

### 3.5. Scanning Fluorescent Microscopy Analysis

**Figure 4:**
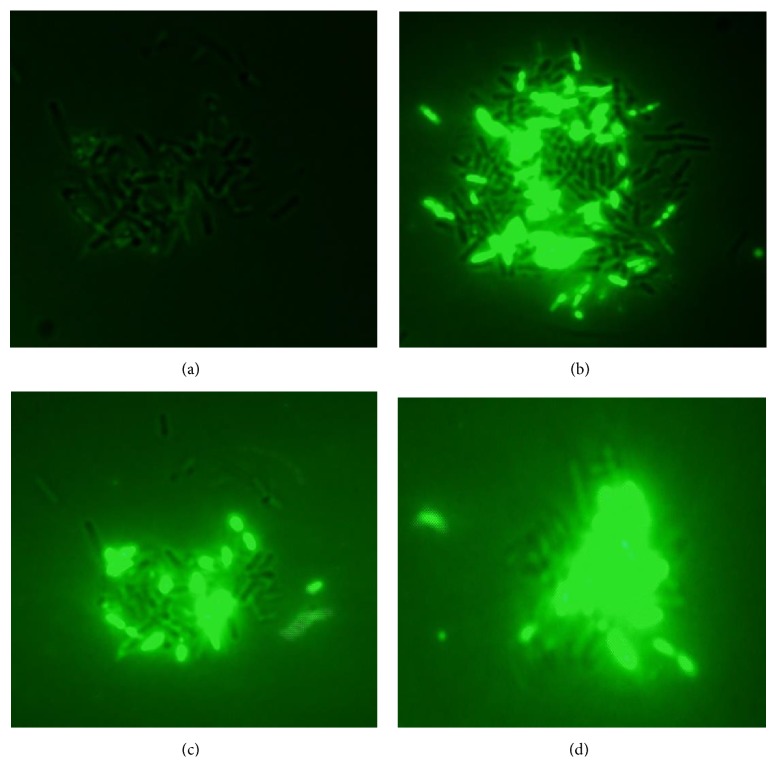
Fluorescence scanning microscopy analysis of* L*.* monocytogenes* ATCC 19117 cells without (a) and treated with bacteriocin BacFL31 for 15 min (b), 30 min (c), and 1 h (d).

In presence of BacFL31, the alteration of* L. monocytogenes* ATCC19117 cells wall permeability and membrane pores formation was confirmed by scanning fluorescent microscopy using as fluorescent probe the SYTOX Green. This molecular probe can only permeate depolarized membrane cells and presents a high affinity for DNA stain [40]. As shown in Figure 4(a), there is no fluorescent signal in the untreated* L. monocytogenes* ATCC19117 cells (negative control) after 1 h of incubation. In presence of BacFL31 at 1 X MIC, the fluorescence intensity increased throughout the incubation time (Figures 4(b)–4(d)). For example, after 15 min of treatment with BacFL31 (Figure 4(b)), almost half of cells exposed to the SYTOX Green dye emit green fluorescence which indicate the damage of approximately 50% of cells. A treatment of 1 h leads to complete damage of cells accompanied by an increase of the fluorescence intensity (Figure 4(d)). Liu et al. [25] reported that destruction of the outer membrane permeability barrier leads to a total or partial dissipation of the proton motive force, causing energy exhaustion and cells death.

### 3.6. Measurement of the Released Extracellular Potassium

**Figure 5:**
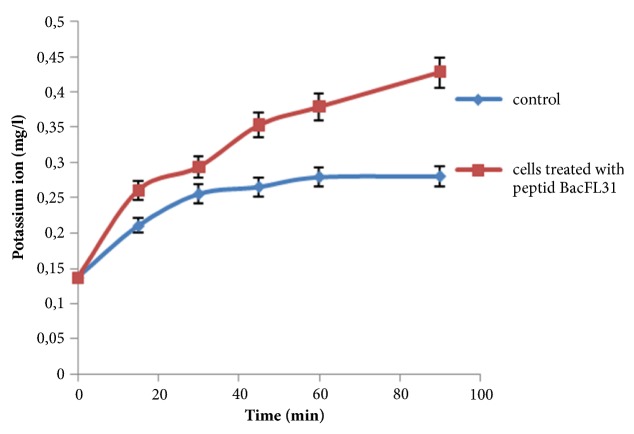
The extracellular levels of potassium ions released by* L. monocytogenes *ATCC19117 cells in presence and in absence of BacFL31.

To determine BacFL31 effect on the integrity of* L. monocytogenes* ATCC19117 cells, the extracellular K^+^ concentration was measured in the culture supernatant of the control and the treated cells. As indicated in Figure 5, BacFL31 caused a significant increase (P<0.05) of the extracellular K^+^ concentration reaching 0.45 mg/L after 60 min of incubation. In contrast, for the control, the levels of the extracellular K^+^ were relatively stable during the test time period (Figure 5). This proves that Bac FL31 increased the permeability of* L. monocytogenes* ATCC19117 membrane cells by pore formation causing leakage of the extracellular potassium ions. It has been reported in previous studies that the peptide F1 causes leakage of potassium ions in* Staphylococcus aureus* [41] and the peptide P7 caused the disruption of S*almonella typhimurium* cells membrane and the leakage of the extracellular K^+^ ions [42].

### 3.7. Measurement of the Transmembrane Electrical Potential (Δ*ψ*)

**Figure 6:**
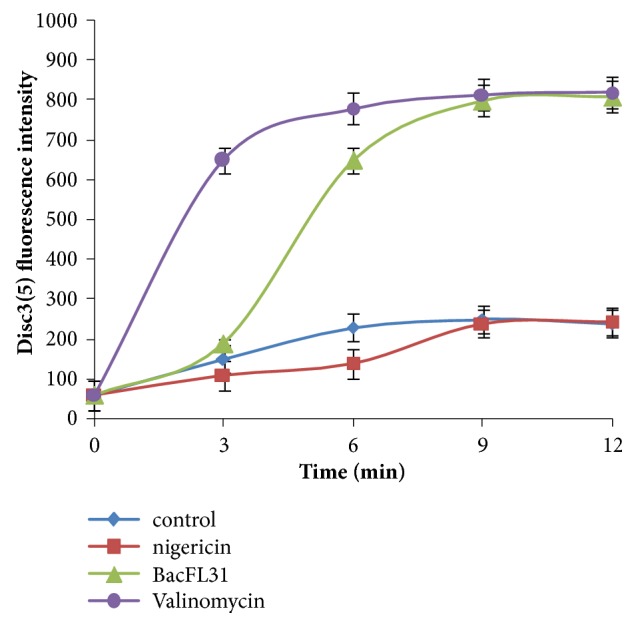
Transmembrane electrical potential (Δ*ψ*) in* L. monocytogenes *ATCC19117 cells. Valinomycin (positive control), nigericin (negative control), and without any addition (control).

In order to assess the effect of BacFL31 on the proton motive force (PMF) of the cytoplasmic membrane of* L. monocytogenes* ATCC19117 cells, we have examined Δ*ψ*. The latter was measured qualitatively with the fluorescent probe 3,3-dipropylthia-dicarbocyanine iodide DISC3(5). The addition of the mobile ion carrier valinomycin (positive control) causes immediately a complete dissipation of the transmembrane electrical potential. In contrast, the cells maintained their Δ*ψ* in the presence of the nigericin (negative control). The addition of BacFL31 causes the depolarization of the cytoplasmic membrane of* L. monocytogenes* ATCC19117 cells like the valinomycin. After 09 min of the addition of BacFL31 (1×MIC) to* L. monocytogenes* ATCC19117 cells, the fluorescence values increased from 60 to 800 (Figure 6). These results proved the role of BacFL31 in the dissipation of the transmembrane electrical potential and membrane depolarization. Probably, BacFL31 could induce permeabilization of the target membrane cells, by forming ion-selective pores which cause dissipation of the proton motive force and depletion of intracellular ATP.

In this regard, several studies reported that Class II bacteriocins induce permeabilization of the target membrane cells by forming ion-selective pores which cause dissipation of the proton motive force and depletion of intracellular ATP such as pentocin 31-1 [43].

## 4. Conclusions

In this study, we showed that the lactic acid strain* E. faecium* FL31 was susceptible to all tested antibiotics, free of hemolytic, gelatinase, DNase, and lipase activities and did not harbor virulence genes. PCR amplification using specific primers indicated that* E. faecium* FL31 possess gene encoding enterocin B. At MIC value, 50 *μ*g/mL, the pure bacteriocin BacFL31 damages cytoplasmic membrane of* L. monocytogenes* ATCC19117, causes the leakage of its intracellular constituents, and leads to the destruction of this pathogenic microorganism.* E. faecium* FL31 and its bacteriocin BacFL31 is a real candidate as natural preservative tool in the food industry for the preservation of food-borne pathogens growth, particularly* L. monocytogenes*, during manipulation and storage of food derivatives.

## Figures and Tables

**Table 1:** Primers used for the screening of virulence genes.

Target gene	Primer (5′-3′)	Ann.temp (°C)	Product size (bp)	References
*asa1*	**ASA11**: GCA CGC TAT TACGAA CTA TGA **ASA12**: TAA GAA AGA ACA TCA CCA CGA	56	375	[15]

*ace*	**ACE-F**: GAA TTG AGC AAA AGT TCA ATC G **ACE-R:** GTC TGT CTT TTC ACT TGT TTC	56	1008	[16]

*efaAfm*	**TE5: **GAC AGA CCC TCA CGA ATA **TE6:** AGT TCA TCA TGC TGT AGT A	54	705	[17]

*esp*	**ESP14F:** AGA TTT CAT CTT TGA TTC TTG G **ESP12R:** AAT TGA TTC TTT AGC ATC TGG	56	510	[15]

*cylB*	**cylB1: **AAG TAC ACT AGT AGA ACT AAG GGA **cylB2: **ACA GTG AAC GAT ATA ACT CGC TAT T	52	2020	[18]

**Table 2:** Primers used for the detection of bacteriocin genes.

Target gene	Primer (5′-3′)	Ann.temp (°C)	Product size (bp)	References
*Ent B*	F: GAA AAT GAT CAC AGA ATG CCT A R: GTT GCA TTT AGA GTA TAC ATT TG	41	159	[19]

*Ent A*	F: GAG ATT TAT CTC CAT AAT CT R: GTA CCA CTC ATA GTG GAA	45	542	[20]

*Ent P*	F: ATG AGA AAA AAA TTA TTT AGT TT R: TTA ATG TCC CAT ACC TGC CAA ACC	41	216	[21]

*Ent L50 A*	F: CCA TGG GAG CAA TCG CAA AA R: AAG CTT AAT GTT TTT TAA TCC ACT CAA T	50	135	[22]

*Ent L50 B*	F: ATG GGA GCA ATC GCA AAA TTA R: TAG CCA TTT TTC AAT TTG ATC	49	252	[23]

**Table 3:** Antibiotics susceptibility of *E. faecium* FL31.

Antibiotics	Concentration (*μ*g/disc)	Sensitivity
Ampicillin	30	S
Streptomycin	10	S
Kanamycin	30	S
Chloramphenicol	30	S
Tetracycline	30	S
Spectinomycin	100	S
Penicillin G	30	S
vancomycin	30	S
oxacillin	5	S
amoxicillin	25	S

S- Sensible

## Data Availability

The* Enterococcus faecium* FL31 strain, the bacteriocin BacFL31 genes detection, the BacFL31 purification and its mode of action against* Listeria monocytogenes* data used to support the findings of this study are included within the article.
